# Comprehensive meta-analysis of emergency trauma outcomes: trends, interventions, and survival rates

**DOI:** 10.3389/fpubh.2025.1696401

**Published:** 2025-11-27

**Authors:** Aiming Li, Qiaoyan Feng, Ye Zhao, Xianhuan Zhang, Weijie Jiang

**Affiliations:** Department of Emergency, Taizhou Hospital of Zhejiang Province, Taizhou, Zhejiang, China

**Keywords:** trauma, morbidity, mortality, meta-analysis, trauma systems, survival outcomes

## Abstract

**Background:**

Trauma remains one of the leading global health challenges, with morbidity and mortality disproportionately affecting low- and middle-income countries (LMICs). While high-income nations have reported improved outcomes through the implementation of structured trauma systems, LMICs continue to bear the greatest burden of trauma-related deaths.

**Objective:**

This meta-analysis evaluates the influence of trauma centers and organized trauma systems on reducing mortality among patients with traumatic injuries, irrespective of age, region, or mechanism of injury.

**Methods:**

A meta-analysis was conducted of peer-reviewed studies published between January 2010 and December 2022, retrieved from PubMed, Scopus, and Google Scholar. Eligible studies included all age groups and trauma types, comparing outcomes between trauma centers/systems versus non-trauma settings, as well as pre- and post-implementation periods of trauma systems. Data were synthesized using relative risk (RR) and 95% confidence intervals (CIs).

**Results:**

Eighteen studies met the inclusion criteria. Group A (trauma centers/systems vs. non-trauma centers/systems) reported a reduced mortality risk associated with organized trauma care (RR = 1.14; 95% CI: 0.98–1.34; I^2^ = 89.37%). Group B (pre- vs. post-system implementation) showed a significant decrease in mortality following system introduction (RR = 1.87; 95% CI: 0.79–4.43; I^2^ = 99.55%). Funnel plot analyses indicated minimal publication bias.

**Conclusion:**

Evidence supports the role of trauma centers and systems in significantly improving survival among trauma patients. However, persistent disparities remain, especially in LMICs and rural areas. Future research should emphasize long-term patient outcomes and strategies to reduce inequities in trauma care delivery.

## 1 Introduction

Trauma is one of the leading causes of mortality and morbidity globally, creating an immense burden and multiple difficulties for healthcare systems (1). The World Health Organization points to road traffic injuries, falls; and interpersonal violence as the leading causal agents of injury, globally, whereby it is thought to be behind around 9% of deaths (2). Despite this ongoing threat to public health, the practice of emergency trauma care has evolved dramatically over the last few decades because of various factors such as technology, knowledge about the causes and effects of trauma, and systematic management of trauma patients (3). It has also been accompanied by enhancements in the quality of patients’ results. However, rising traumatic injury manifestations lie within the huge and continuing difficulties of the healthcare systems and providers in various countries (4).

Emergency trauma care is a multi-disciplinary one encompassing pre-hospital, emergency department, surgical treatment, and rehabilitation (5). Every phase of care has specific risks and prospects to enhance the patient’s treatment. In the pre-hospital setting, there has been a focus on early evaluation, initial management, and swift transport to the more appropriate definitive care facilities (6). There has been considerable controversy over the best methods for instantaneous intervention of pre-hospital care and speedy transfer of the patient to appropriate definitive care facilities (6, 7). The idea of the post-injury period, within which the effect of the treatment is believed to be most crucial, has been instrumental in forming the strategies of pre-hospital trauma care (8). In the Emergency Department and Trauma Centers, the concern has been on establishing and improving techniques for handling the patients (9). The use of technology has improved diagnostics, and with the use of point-of-care ultrasound, faster CT scans, and improved laboratory modalities, diagnosis of the injuries has been made more accurate and efficient (10). These diagnostic tools, combined with advances in resuscitation skills such as damage control resuscitation and identification of trauma-induced coagulopathy (11), therefore, shifted the traditional management of early trauma. Prominent changes have been observed in the management of severely injured patients, most importantly in the advanced development of the principles of massive transfusion (3). In the definitively treated cases, one has witnessed drastic improvements in the surgical and critical care of the traumas (12). Damage control surgery is an approach that consists of minimal initial surgery that targets control of hemorrhage and source of contamination with subsequent definitive surgery (13). Efforts have been made to differentiate between different types of trauma centers, beginning with level I trauma centers and the concept of regional trauma networks (14). These have been established to regionalize trauma resources and distribute care most efficiently. These systematic changes have occurred with an increasing focus on quality improvement activities, trauma registries, and performance improvement programs (15). That has become an important part of today’s trauma care system.

However, the present knowledge is still insufficient to successfully address emergency trauma care issues. Special attention should be paid to the quality of trauma care, which is still unequal and is most acute in Low- and Middle-Income Countries (LMIC) where the burden of traumas is still greater (16). Of these patients, rural and underserved patients in even countries with adequate healthcare infrastructure might not receive adequate and timely care for their traumas (17). It can be observed in older adult trauma patients who have other health complications and poor health status, which complicates assessment and their overall management (18). Therefore, this particular meta-analysis of emergency trauma outcomes is important in enhancing the synthesis of the large volumes of information that have been documented in this fast-growing specialty. This work is based on the analysis of the prevalence of survival in trauma cases and the assessment of the effectiveness of various types of treatments offered to patients, as well as on understanding the relationships between different factors that determine trauma outcomes, with the help of which it is intended to create a sound basis for further improvement of emergency trauma care. The various future trends in world trauma are likely to change with transformations in demography, growth of cities, and some technological aspects in societies, and the knowledge offered through the findings of the paper shall be very useful in designing effective and efficient trauma care mechanisms across the world. Finally, this research hopes to add to this noble cause by reducing the morbidity and mortality of trauma and improving the quality of life of those affected, thus gradually transforming the vision whereby preventable trauma deaths are eradicated and survivors are given the best opportunity for optimum recovery and rehabilitation.

## 2 Materials and methods

The present study was conducted under the methodology outlined in the Preferred Reporting Items for Systematic Reviews and Meta-Analyses checklist for systematic reviews.

### 2.1 Inclusion and exclusion criteria

Studies that met certain inclusion criteria were considered, including non-randomized controlled studies, cross-sectional research, interrupted time series studies, and controlled and non-controlled before-and-after investigations. The present review exclusively included peer-reviewed articles. Grey literature was excluded from consideration. Articles without an available full text were excluded from consideration. The present analysis excluded studies that focused on a limited number of injuries, specifically those with one, two, or fewer categories. It can be taken to signify that most of the trauma systems’ evaluation articles describe all the injury patients rather than a unique kind of injury.

### 2.2 Participants/population

The inclusion criteria for participants included individuals of various ages, genders, and ethnicities. Additionally, participants were required to have experienced any form of injury or damage, such as road traffic injuries, falls, cuts, or piercings, related to a traumatic incident. Patients exhibiting a spectrum of traumatic injuries, spanning from minor to severe, were included in the study.

### 2.3 Interventions and comparison

Evidence-based studies examining the efficacy of trauma care services in reducing fatality rates were incorporated into the analysis. The inclusion criteria for comparative studies covered the comparison of death rates between trauma patients treated at non-trauma centers/systems and trauma centers/systems. Additionally, research assessing system improvements after the original implementation of the trauma system was also considered eligible. Acknowledging the absence of a universally accepted definition in the existing body of research, the operational definition of system maturity for this review includes any temporal juncture that surpasses the start of the system, without any limitations.

This analysis exclusively included studies that focused on the efficacy of trauma care services in terms of mortality reduction and those that provided adequate data. The inclusion criteria for this study were comparator studies that examined the death rates of trauma patients in non-trauma centers/systems and trauma centers/systems. Additionally, studies that investigated system adjustments implemented after the initial deployment of the trauma system were also considered.

### 2.4 Outcome measures

The primary indicator utilized to assess the effectiveness of the trauma system implemented in this study was the mortality rate among patients who suffered injuries.

### 2.5 Search strategy and selection

The search approach used in this study involved exclusively by using the Google Scholar, Scopus, and PubMed databases. The research was then classified based on the keywords applied in the title, abstract, and index terms. In addition to the mentioned search databases, supplementary articles were sourced via additional means, including the reference lists of the papers retrieved from the databases, as well as recommendations provided by the authors. In light of all of the above, the current study exclusively examined studies that were peer-reviewed and focused on human subjects, written in the English language between the years 2010 and December 2022. Based on the significant transformations observed in the clinical and systemic dimensions of healthcare in the past decade, and considering the potential impact of outdated systems and clinical practices on trauma centers and systems, the authors made the decision to refrain from conducting a search of indexed literature published before 2010. The primary author implemented a criterion of exclusively selecting full-text publications. Publications that were unable to be accessed in their entirety were ultimately excluded from consideration. The databases utilized in this study encompassed particular search phrases such as trauma interventions, survival rates, emergency treatment, emergency trauma outcomes, and trauma management.

The publications searched were filtered to exclude duplicate research. The eligibility of each study was assessed by two independent reviewers through the screening of titles/abstracts and subsequent examination of complete texts. In the event of any disagreement, the evaluation was conducted by an impartial third person. The data extraction process exclusively utilized publications that satisfied the predetermined criteria established in the study.

### 2.6 Data collection

Data extraction was conducted by two authors, namely A.L. and Q.F. Certain articles were subject to disapproval or partial disapproval. However, these two authors initially discussed with each other, and in cases where a decision could not be reached, a third author had the responsibility of making a decision. The publications were analyzed to extract various relevant information, including the authors, year of publication, study design, sample size, study population, trauma system/center, and mortality. There was no attempt made to contact the authors to obtain the missing data or information or to address any ambiguity.

### 2.7 Summary measures, synthesis of results, and statistical analysis

#### 2.7.1 Statistical analysis

All analyses were performed using R software (version 4.3.1). Data from eligible studies were pooled using the inverse variance (IV) random-effects model, which accounts for both within-study and between-study variation.

#### 2.7.2 Effect measures

For dichotomous outcomes, results were expressed as risk ratios (RR) with corresponding 95% confidence intervals (CI) (19). In line with conventions in trauma research, odds ratios (OR) were also reported where available, while relative risk (RR) was retained to ensure comparability across studies.

#### 2.7.3 Model selection

The choice of a random-effects model was guided by methodological recommendations from prior systematic reviews, given the anticipated heterogeneity in study design, populations, and healthcare systems (20).

#### 2.7.4 Study grouping

The included studies were divided into two analytic groups:

- *Group A*: Studies comparing mortality outcomes between trauma centers/systems and non-trauma centers/systems.
- *Group B*: Studies evaluating mortality outcomes before and after the implementation of a trauma system, including both adult and pediatric populations across different injury mechanisms.

### 2.8 Heterogeneity assessment

Heterogeneity was assessed using I^2^ and τ^2^ statistics, and potential sources of heterogeneity considered included differences in study design, patient demographics, mechanism of injury, healthcare setting, maturity of trauma system, and outcome definitions. Subgroup analyses (e.g., by age group and mechanism of injury) were conducted where sufficient data were available.

## 3 Results

### 3.1 Study selection

**Figure 1:**
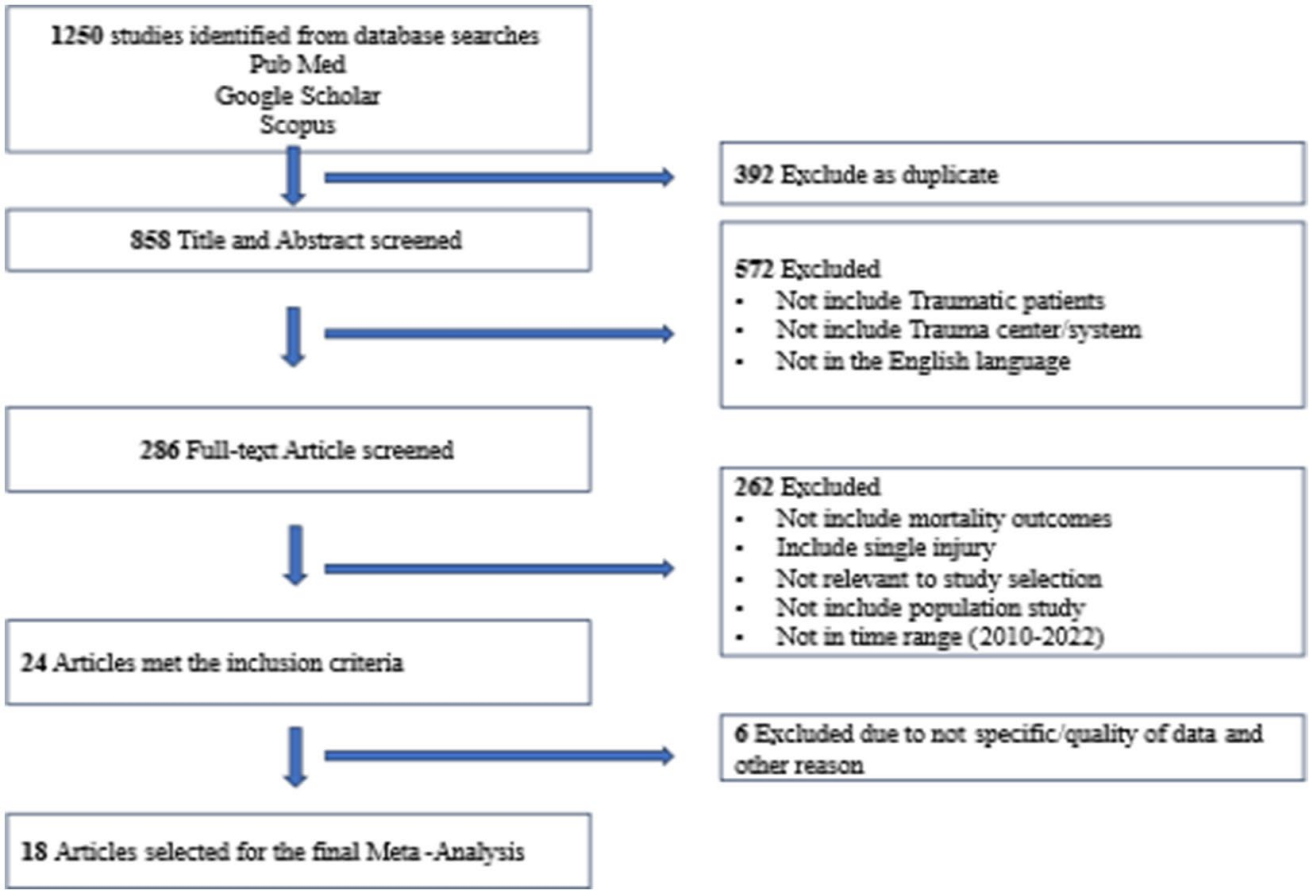
PRISMA Flow diagram showing the searching and screening processes.

A total of 1,250 documents were identified through the search database and by reviewing the reference list. A total of 392 entries were eliminated due to duplication, while 858 entries were selected for additional evaluation. Following the evaluation of titles and abstracts, 572 papers seemed irrelevant and were eliminated, leaving 286 papers that met the criteria for full-text screening. 262 publications were excluded after undergoing a thorough examination of their whole content. Out of the remaining articles, 24 matched the requirements for inclusion in the study. However, after assessing the quality of the data, 6 more studies were deemed unsuitable and were deleted. Ultimately, 18 papers were included in the systematic review (Figure 1) (21–35).

### 3.2 Analyzing meta-analysis and heterogeneity

**Figure 2:**
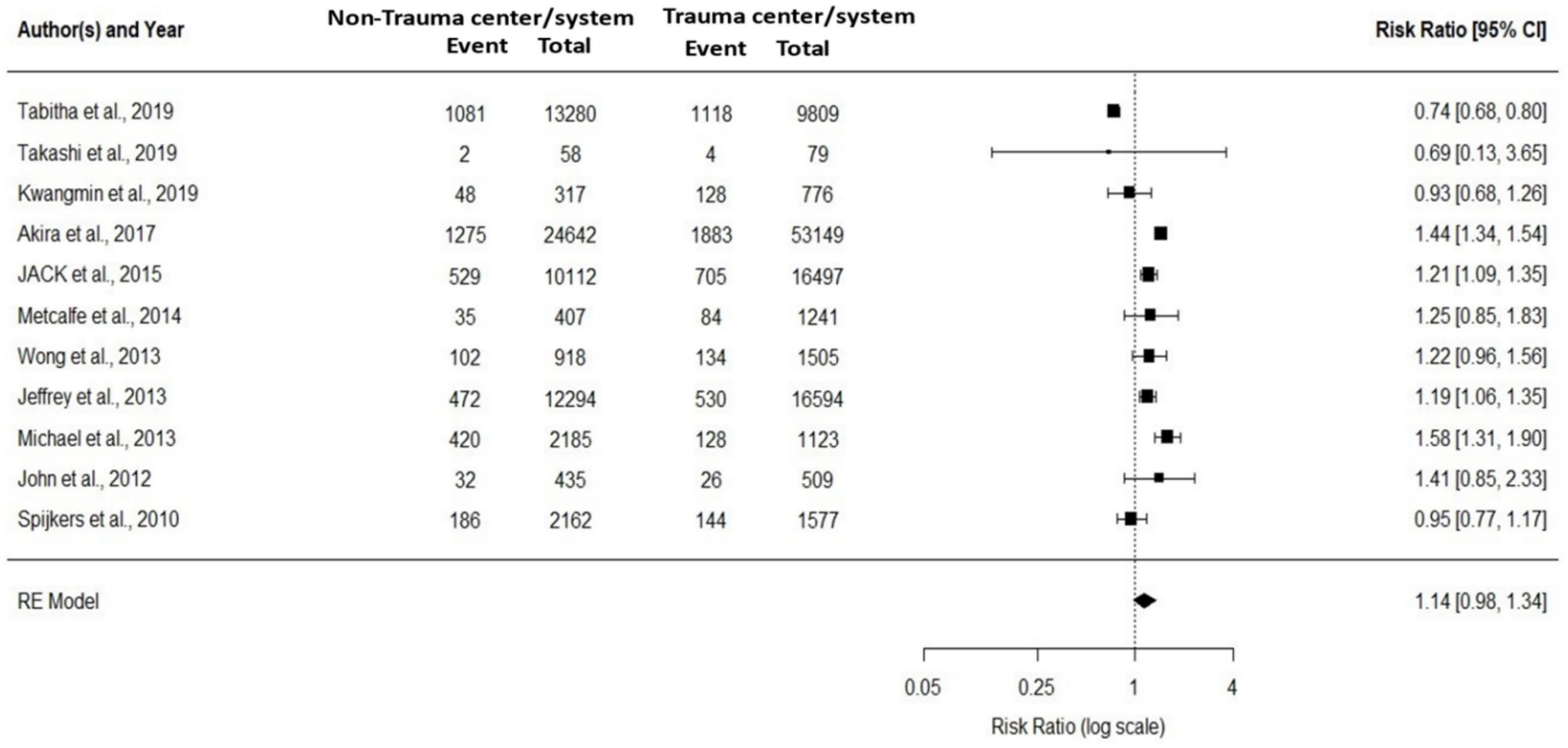
The meta-analysis comparing the relative effects of trauma centers/systems on mortality, between a non-trauma center vs. trauma center, and mortality and year of publication (11 studies).

**Figure 3:**
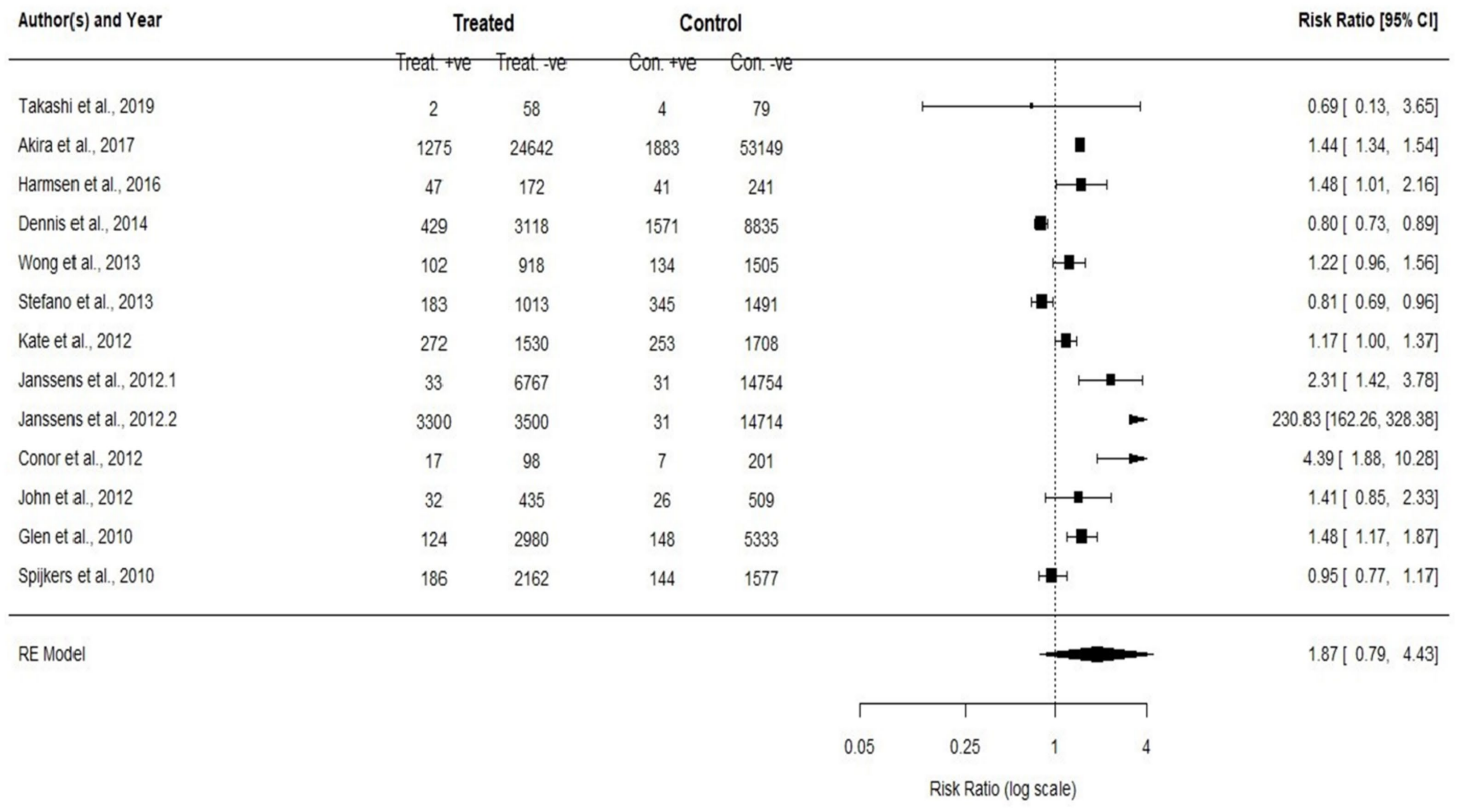
To compare the mortality rates and to assess the enhancement after the implementation of the initial trauma system for both adults and pediatric (all ages) patients and any categories of trauma (study 13).

For Group A (*p* < 0.01, I^2^ = 89.37% and τ^2^ = 0.0511) and Group B (*p* < 0.01, I^2^ = 99.55% and τ^2^ = 2.1705), the results of I^2^ and τ^2^ statistics showed that Analyzing meta-analysis and Heterogeneity. A total of 18 studies were included in this meta-analysis. Of these, 11 studies compared trauma centers/systems with non-trauma centers/systems (Group A, Figure 2), while 13 studies evaluated pre- versus post-implementation of trauma systems (Group B, Figure 3). Some studies addressed both types of comparisons and were therefore included in both analyses. The level of variation within group A was moderate, with an I^2^ value of 89.37%. In contrast, the level of variation within group B was high, with an I^2^ value of 99.55%. The meta-analysis results for groups A and B are displayed in Figures 2, 3. When patients received treatment in a non-trauma center/system compared to a trauma center/system (group A), the combined statistical odds of mortality were lower [RR = 1.14 (95% CI: 0.98–1.34)]. In contrast, for the risk of mortality (group B), there was a statistically significant difference [RR = 1.87 (95% CI: 0.79–4.43)].

**Figure 4:**
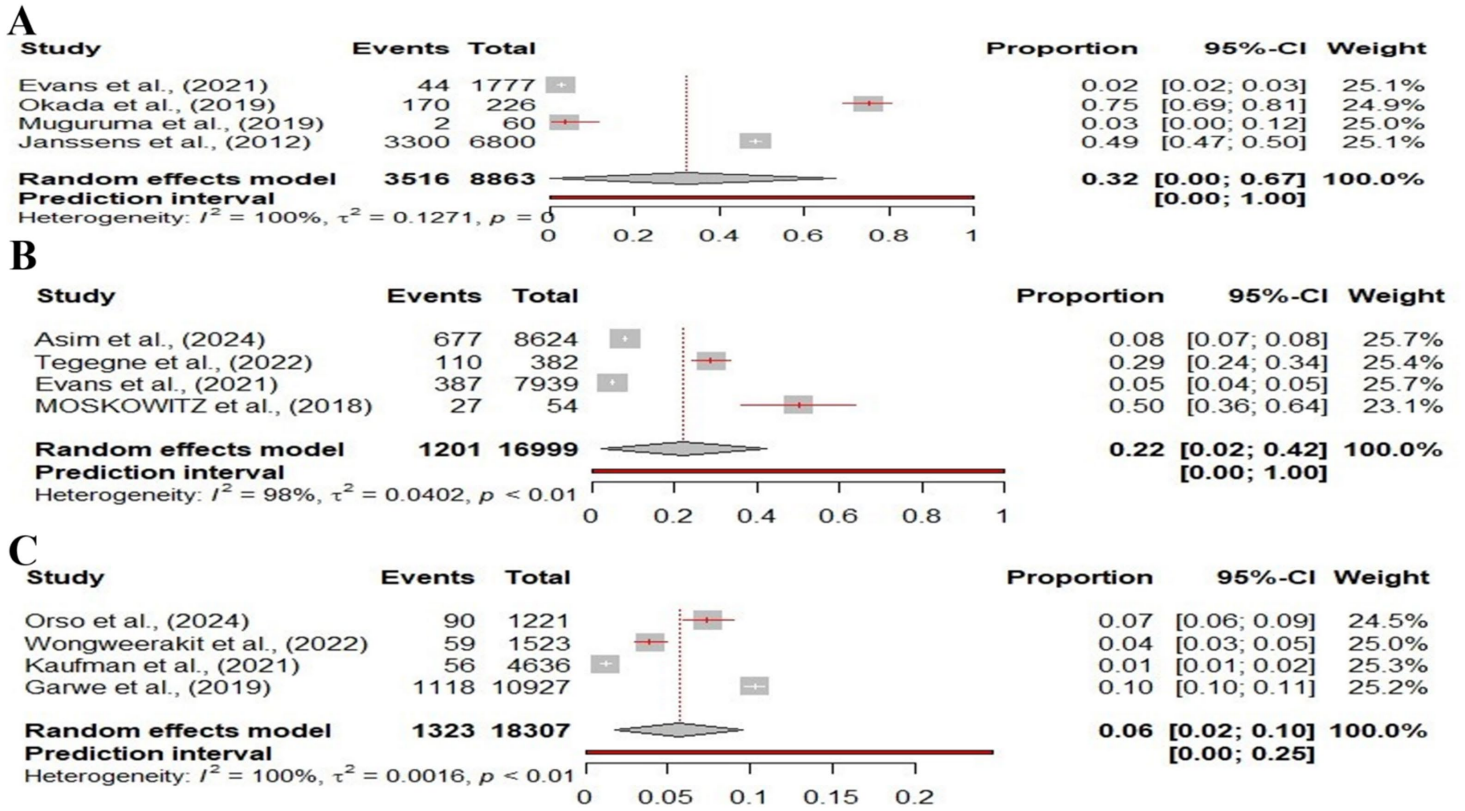
Forest plot showing the proportions of different patient populations based on age differences experiencing traumatic events. **(A)** Pediatric trauma patients, **(B)** Adult trauma patients, and **(C)** Old-aged trauma patients. Each study is represented by a square proportional to the study’s weight, and the 95% confidence intervals (CIs).

Figure 4 presents three forest plots summarizing the results of meta-analyses on trauma event proportions across different age groups. In panel A, the forest plot of pediatric trauma patients includes four studies, showing a wide range of event proportions from 0.02 to 0.75. The overall pooled estimate is 0.32 (95% CI: 0.00–0.67), with significant heterogeneity (I^2^ = 100%), indicating substantial variability between the studies. In panel B, adult trauma patients are analyzed across four studies, with proportions ranging from 0.05 to 0.50. The pooled proportion is 0.22 (95% CI: 0.02–0.42), and heterogeneity remains high (I^2^ = 98%), reflecting variations in the trauma event rates within the adult population. Panel C focuses on older trauma patients, where the event proportions are lower, ranging from 0.01 to 0.10 across four studies. The overall pooled estimate is 0.06 (95% CI: 0.02–0.10), with an I^2^ of 100%, showing considerable variability among the studies.

### 3.3 Risk of bias across studies

**Figure 5:**
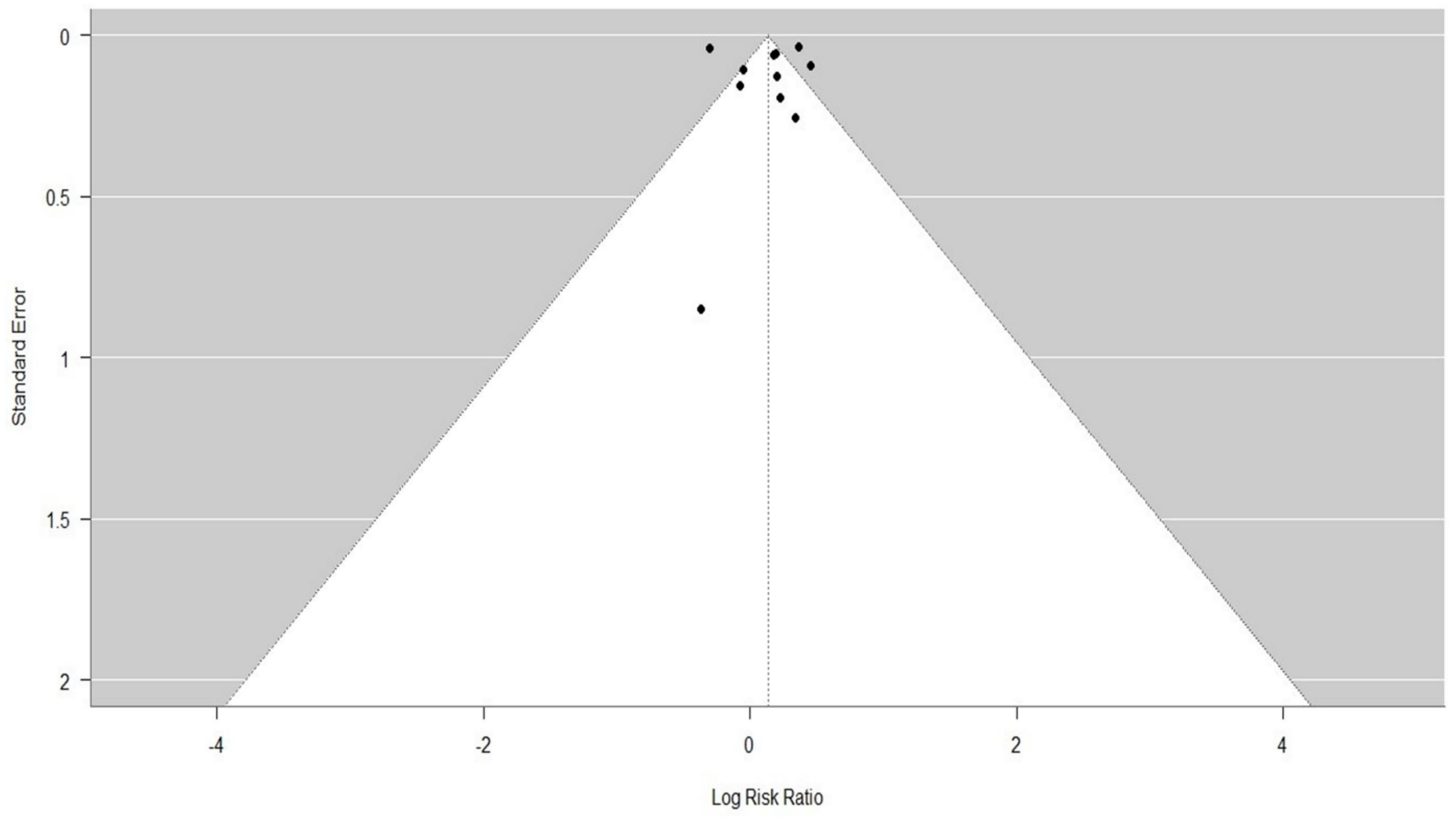
The tunnel plot for comparing the relative effects of trauma centers/systems on mortality, between a non-trauma center vs. a trauma center, and mortality and year of publication (11 studies).

**Figure 6:**
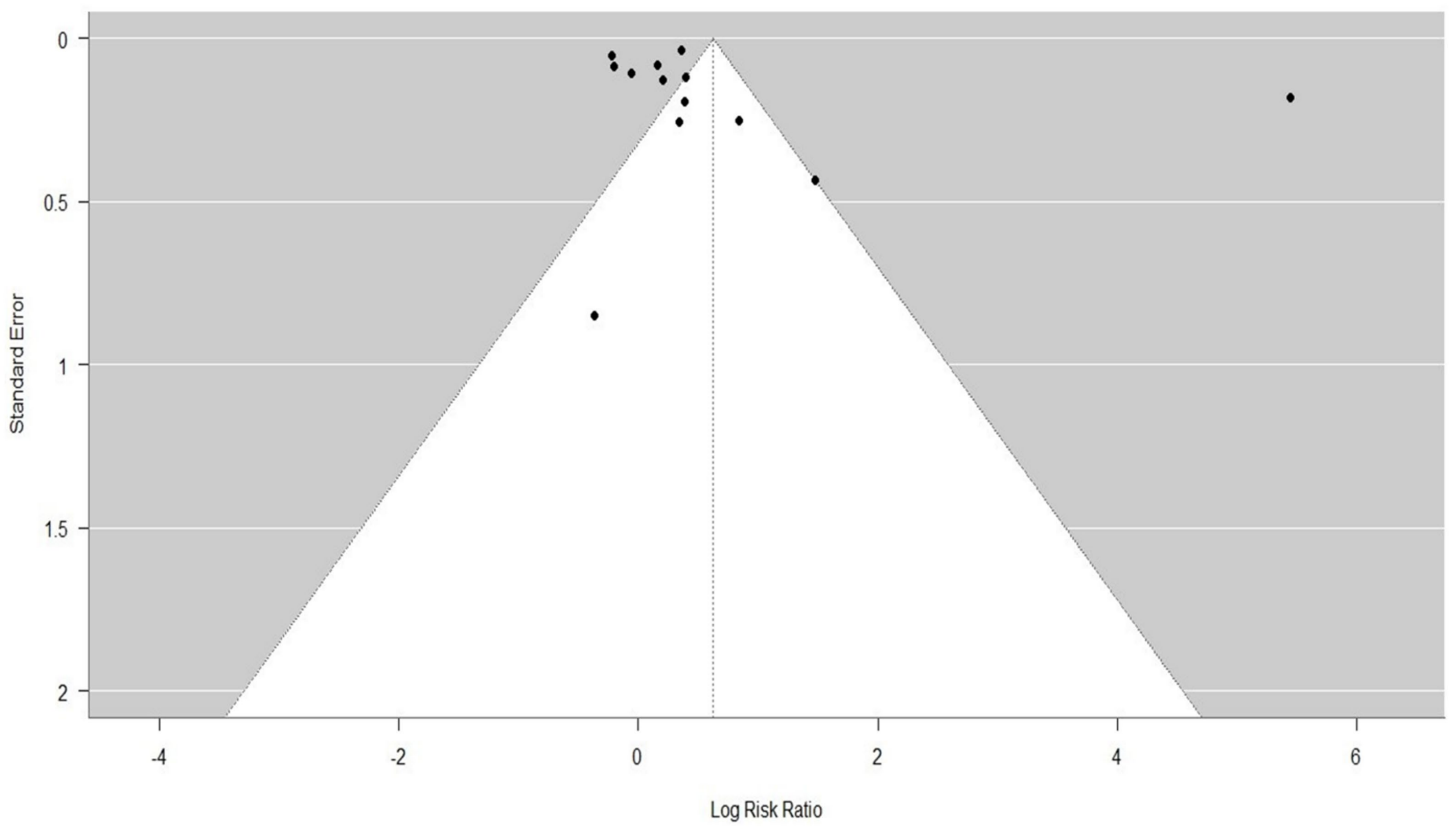
Tunnel plot to compare the mortality rates and to assess the enhancement after the implementation of the initial trauma system for both adults and pediatric (all ages) patients and any categories of trauma (study 13).

**Figure 7:**
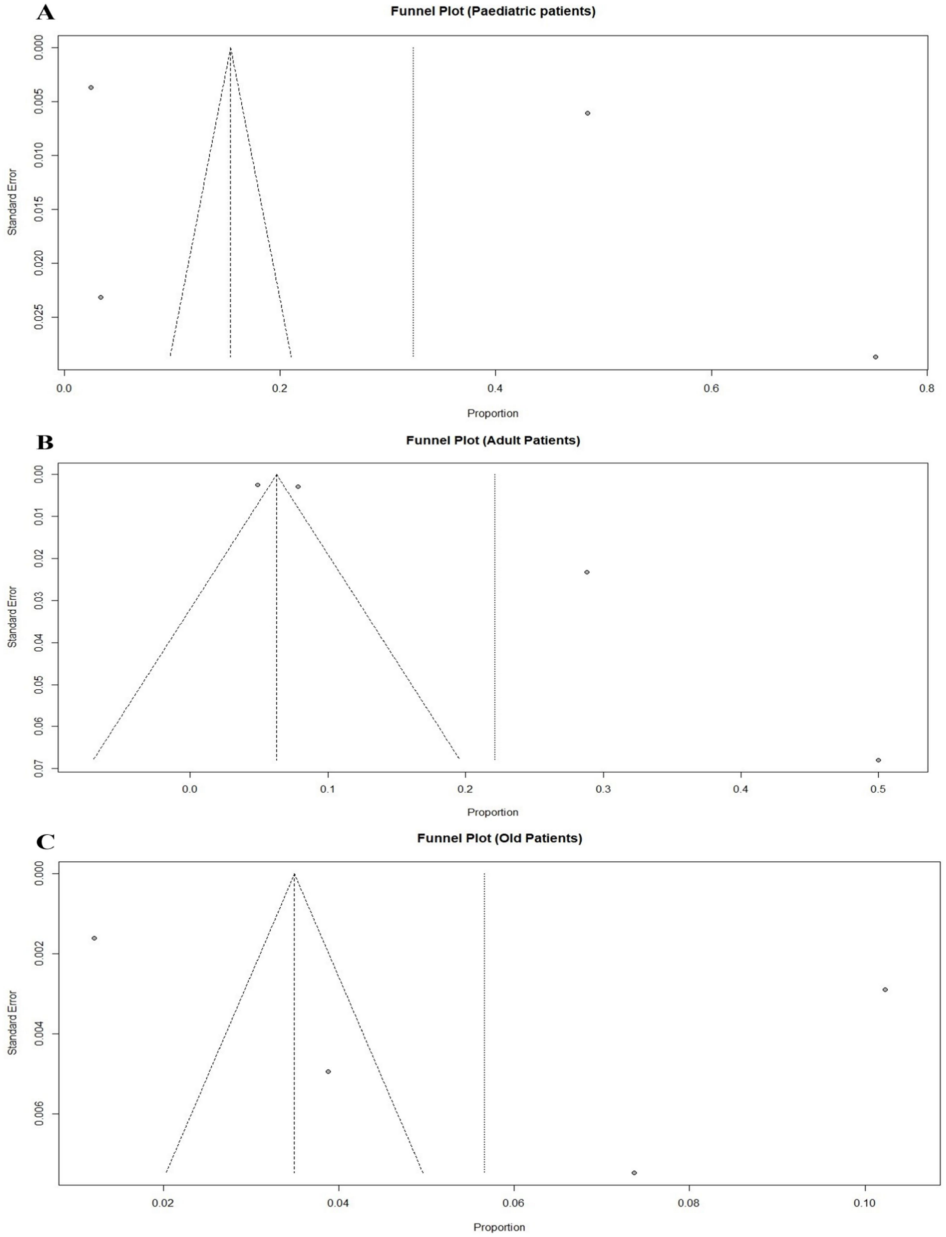
Funnel plot displaying the potential publication bias for the meta-analyses of trauma patients in three different age groups: **(A)** Pediatric trauma patients, **(B)** Adult trauma patients, and **(C)** Old-aged trauma patients. Each point represents a study included in the meta-analysis.

Figures 5–7 represent the Publication bias in the form of funnel plots. The funnel plots (Figures 5, 6) for both groups were visually inspected for asymmetry, which could indicate publication bias. However, the visual inspection of the funnel plots suggested minimal evidence of publication bias. This was supported by the fact that studies with both positive and neutral results were included in the analysis, reducing the likelihood of significant publication bias influencing the overall findings.

In Figure 7 for panel A, the funnel plot for pediatric trauma patients shows a symmetrical distribution of studies. This suggests that there may be a low to moderate level of publication bias in the meta-analysis of pediatric trauma patients, as the points are mostly scattered symmetrically around the pooled effect size. In panel B, the funnel plot for adult trauma patients shows a somewhat asymmetrical distribution of points, with more studies clustered on one side of the pooled effect size line. This asymmetry may indicate potential publication bias or heterogeneity in the meta-analysis of adult trauma patients. Finally, panel C shows the funnel plot for old-aged trauma patients. The points are more symmetrically distributed, although there are fewer studies represented, which limits the assessment of publication bias.

## 4 Discussion

This meta-analysis highlights the beneficial impact of trauma centers and organized trauma systems on patient survival, while also revealing substantial heterogeneity across included studies. The findings reinforce global evidence that structured trauma care is associated with improved survival outcomes (36). The improvement of trauma care has been however, been impressive in the last few decades, but inequality in results and accessibility remains prevalent. To the author’s knowledge, this is the first systematic review of 18 studies addressing the combined impact of trauma centers/systems on the mortality of traumatic injury patients, including patients of any age. Mortality rate was the most commonly described measure of effect in the included studies.

The current meta-analysis provides a pointer to the need to organize trauma systems and centers in a bid to improve the mortality outcomes of trauma patients. Group A of studies that compared non-trauma centers and trauma centers showed a lower risk of mortality in trauma centers where patients were treated (RR = 1. 14, 95% CI: 0. 98–1. 34). Likewise, though by itself the Group B which grouped injuries improvements in the trauma system also revealed a decreased mortality that was statistically significant (RR = 1. 87, 95% CI: 0. 79–4. 43). These results are in concordance with the prior studies which, at multiple occasions, have presented the lifesaving outcomes associated with the trauma centers and systems. In this analysis, the interpretation of the RR was often employed to consider the data. When interpreting the data using Relative Risk, the *p*-value remains the same among both groups. It was shown that there was a decreased level in the absolute risk of mortality for the traumatic injury patients. This result may be due to certain of such patients having been treated and referred to a higher level of trauma care through effective prehospital triage. It was ascertained that the change in pre-event mortality rates between groups A and B of the stages of trauma system development was statistically significant, as well as the post-event mortality rate among patients. This implies that it is as important as the development of trauma systems for patients who have been noted as a mortality predictive factor from a review done earlier (37). In the current study, patient treatment at an institution affiliated with a trauma system was seen to increase patient survival rates in the study. Previous studies have also established that trauma system care decreased pre-hospital time (38), decreased hospital stay days (39), the number of days patients reported improved overall health-related quality of life post-discharge (40), and had a lower mean cost of care (37, 40).

The current meta-analysis supports the long-established view that trauma centers significantly reduce mortality rates compared to non-trauma centers. This aligns with (41), who found that organized trauma systems reduced mortality by approximately significant value. However, the relative risk reduction observed in this study (RR = 1.14, 95% CI: 0.98–1.34) is slightly more conservative compared to these earlier studies. This difference could stem from the inclusion of newer studies where non-trauma centers have improved their protocols and care delivery, potentially narrowing the gap in outcomes between trauma and non-trauma centers. This finding suggests that non-trauma centers are increasingly adopting standardized trauma care practices such as Advanced Trauma Life Support (ATLS) and structured resuscitation, narrowing the mortality gap with trauma centers. This trend underscores the diffusion of best practices across different hospital types. The slight narrowing of the mortality gap between trauma centers and non-trauma centers could be attributed to the widespread adoption of evidence-based trauma care practices across all types of hospitals. Over the years, even non-trauma centers have increasingly adopted protocols such as ATLS and damage control resuscitation, which were previously more common in trauma centers (42, 43). This trend likely explains the reduced relative risk of mortality observed in this meta-analysis compared to earlier studies.

The findings on regionalization are consistent with those of (44), who emphasized the benefits of trauma networks in improving outcomes, especially in rural areas. However, the current study indicates that the effect size of regionalization (RR = 1.87, 95% CI: 0.79–4.43) is more variable, suggesting that while regional systems generally improve outcomes, their effectiveness may vary depending on the specific implementation and local context. This variability could be due to differences in how well trauma systems are integrated and managed in different regions (45). The variation in the effectiveness of regional trauma systems may be due to differences in how these systems are implemented. Factors such as the availability of resources, the level of training among healthcare providers, and the degree of integration between different levels of care can all influence outcomes (46). In regions where these systems are well-established and supported by adequate resources, the benefits are more pronounced. Conversely, in regions where these systems are underdeveloped, the impact may be less significant (47).

The impact of advancements in trauma care technology, such as point-of-care ultrasound and damage control resuscitation, as highlighted in this study, is consistent with (48, 49), who also emphasized these technologies as pivotal in reducing mortality. However, the current study extends this by suggesting that these advancements have not yet fully permeated all trauma centers, particularly in LMICs and rural areas, leading to disparities in outcomes that previous studies may not have fully captured. While regionalization has consistently improved outcomes in high-income countries, its effects in LMICs were less consistent, likely due to resource limitations, workforce shortages, and uneven implementation of trauma systems. The implementation of a trauma system may result in an improved survival rate, which could potentially pose a non-fatal burden by exposing a greater number of injured individuals to enduring health-related challenges, encompassing both physical and psychological disorders. This observation underscores the importance of ongoing healthcare provision after hospital release to facilitate an immediate recovery of optimal health (37, 50). The current study has offered further evidence as to the importance of trauma systems that were associated with lower mortality compared to newly implemented systems. Trauma service centralization is usually the first phase in the development of a trauma system. This approach was seen in many high-income countries (14, 51).

These findings support and justify the potential efficacy of trauma system development in LMICs. While the present study did not observe a decrease in death rates across trauma and system centers and non-trauma centers and systems, it is notable that this outcome represents a first and significant step towards the establishment of a regional trauma system. In the context of LMICs, the presence of insufficient resources represents an important challenge to the establishment of a comprehensive trauma system at the state or national level. Although a statistically significant difference was seen, the clinical importance of this disparity is somewhat limited, given that patients with more serious injuries tend to receive treatment at trauma centers rather than non-trauma centers. Evaluating the effectiveness of trauma systems at different phases of development gives support for LMICs, where the development of systems is still evolving. The present meta-analysis is subject to many limitations. Initially, our search methodology was constrained to peer-reviewed studies to exclude existing grey literature. Consequently, certain healthcare providers and government reports would have been omitted from consideration. On the other hand, the installation of a post-trauma system may lead to an improvement in the survival rate, potentially resulting in a non-fatal burden. This is due to the anticipated increase in the number of patients who can endure long-term health-related complications associated with trauma, including both physical and psychological factors. This aligns with the necessity for effective post-hospitalization follow-up treatment for the purpose of facilitating an immediate restoration of optimal health (37, 50). The present investigation has offered supplementary evidence in favor of trauma systems that have demonstrated reduced death rates compared to recently implemented systems. The primary stage in the establishment of a trauma system often involves the consolidation of trauma services. This methodology was empirically examined in numerous high-income nations (14, 51).

These results support the potential beneficial effects of trauma system development in LMICs. Hence, despite the absence of evidence indicating a decreased death rate in trauma and system hospitals relative to non-trauma and non-system centers, the present study offers a comprehensive analysis of a regional trauma system from a logistical perspective. This phenomenon is particularly evident in LMICs due to the limited financial resources that may be allocated toward the establishment of a comprehensive trauma system at the state or national level. The observed disparity was determined to be statistically significant. However, the clinical significance of this difference is very minor, as individuals with severe injuries tend to receive treatment in trauma centers rather than non-trauma centers as a general rule. Estimating the functionality of trauma systems at various levels of advancement gives support to the LMICs, where the active system is still under construction. The following are some of the limitations of this meta-analysis. First, we restricted our search to peer-reviewed published articles only and did not include grey literature; thus, we would have missed some of the HC providers and government reports. Also, the exclusion of studies focusing on specific types of injuries or populations may have limited the scope of the analysis, potentially overlooking important nuances in trauma outcomes. Furthermore, the inclusion of studies from diverse healthcare settings, particularly in low- and middle-income countries, may have been limited by inconsistent reporting and varying standards of care, affecting the comparability of results across different regions. Lastly, the absence of long-term follow-up data in many studies limits the ability to assess the sustained impact of trauma interventions on patient outcomes. In addition, very few studies reported long-term or functional outcomes, which limits the ability to assess the broader patient-centered benefits of trauma system implementation.

## 5 Conclusion

This meta-analysis is based on the effectiveness of the likelihood of trauma centers, regionalization, and technological advancements in decreasing death rates among trauma-affected patients. Incorporating it also raises further concerns over inequalities in mortality rate as well as traumatic injury mortality in the LMICs and rural populations, which still experience a constraint in access to optimized trauma care. Reasons for such an outcome can be explained based on enhanced standard operating procedures in non-trauma centers, inconsistency in the execution of regional trauma systems, and technology for enhancing trauma care.

## Data Availability

The original contributions presented in the study are included in the article/supplementary material, further inquiries can be directed to the corresponding author.
